# Composition and Dynamics of the Black Sea Benthopelagic Plankton and Its Contribution to the Near-Shore Plankton Communities

**DOI:** 10.1371/journal.pone.0099595

**Published:** 2014-06-19

**Authors:** Alexander L. Vereshchaka, Ludmila L. Anokhina

**Affiliations:** 1 P.P. Shirshov Institute of Oceanology of Russian Academy of Sciences, Laboratory of structure and dynamics of plankton communities, Russia, Moscow; 2 Moscow State University for Geodesy and Cartography, Department of Applied Ecology, Russia, Moscow; Dauphin Island Sea Lab, United States of America

## Abstract

At a shallow (7 m) near-shore sampling site in the Black Sea we analyzed composition, abundance, and biomass of benthopelagic organisms and the contribution these animals make to the total plankton. The site was monitored across several years (1996–2001; 2006–2007) whilst for 1999–2000 the seasonal variations were analysed. A total of 321 samples from Golubaja Bay near Novorossiysk (44°34′31.04″ N, 37°58′45.11″ E) in 1996–2007 were taken with a Judey net. The benthopelagic fauna was represented by 69 taxa, a diversity comparable to similar shelf areas. The benthopelagic component played an important role in near-shore plankton communities in the Black Sea accounting for 50% of the total zooplankton biomass at night during all seasons. Abundance and biomass of the benthopelagic animals showed seasonal fluctuations, the highest biomass being recorded during winter (>75% of the total zooplankton biomass) and early spring due to large amphipods, whilst the highest abundances occur during late summer because of numerous young stages of various taxa. Amphipods, mysids, and decapods are the main contributors to the plankton biomass and abundances. Both night and daytime samples are strongly recommended for the adequate description of the near-shore plankton communities.

## Introduction

Interaction between ocean waters and the sea-floor causes a mixed benthic boundary layer which has proved to be ubiquitous in the World Ocean [1]. This layer contains the principal biotope where pelagic and benthic communities interact, and occurs from shelf depths to the abysss [2]. The benthic boundary layer harbours a diverse benthopelagic fauna both in the deep sea [3]–[7], above seamounts and continental slopes [8]–[10], and the shelves [11]–[14] including sublittoral macrophytes [15], coral reefs [16]–[20], mangroves [21]–[22], estuaries [23]–[25], and drifting algae [26]–[28].

The benthic boundary layer is inhabited by three principal ecological groups: pelagic plankton (holoplankton), meroplankton (larvae of benthic animals), and benthopelagic plankton (also called demersal plankton, hyper- or supra-benthos) [2]. Holoplankton are dominated by copepods and chaetognaths, whilst meroplankton are the larvae of benthic animals,. The terms “pelagic” and “benthopelagic” are ecological, not systematic units; similar species of the same genus may refer either to pelagic or to benthopelagic animals, for example, decapods of the genera *Oplophorus, Sergia, Sergestes* – [2], [10], [29]–[30]. One of challenges of benthopelagic fauna research is to develop a sampling method to separate benthopelagic and pelagic plankton near the sea-floor. The first objective of this paper is to describe and test such a method, and to create a list of truly benthopelagic species.

The benthopelagic plankton migrate between water column and seabed to feed, hide or reproduce. Over shelves, continental slopes, and seamounts benthopelagic animals migrate diurnally, ascending to the water column at night and descending to the bottom/in the near-bottom layer in daylight hours. The diurnal vertical migration is one of the most important biological patterns of the benthopelagic fauna. These migrations affect whole populations and whole benthopelagic and pelagic communities [2], [31]. Diurnal migrations involve major selective advantages for the participants, such as capacity to avoid diurnal visual predators, the ability to decrease metabolic consumption in deeper colder waters during the day, the use of other habitats during the pelagic phase, which may provide additional food resources, aids for species dispersal, and increasing the possibility of meeting a sexual partner [31]. As the diurnal migrations are an essential part of the biology of the benthopelagic fauna, the second objective of this paper is to analyse diurnal pattern of the near-shore plankton communities of the Black Sea.

As other temperate areas, the Black Sea ecosystems are exposed to seasonal environmental changes. Benthopelagic communities were surveyed at different seasons [8]–[28], but no continuous record of seasonal observations across a one-year period has been made previously. Here we present such a record and analyze seasonal variability of the benthopelagic plankton, as the third objective of this study.

The shelves are inhabited by a specific benthopelagic fauna. In most cases, however, studies were focused on the benthopelagic animals as such and no attempt was made to compare or estimate the contribution of pelagic and benthopelagic animals. Several estimations were made for the coral reefs and coastal areas of the Northwest Europe, but no information concerning ecosystems of the closed/semiclosed areas like the Black Sea is available. Such studies are, however, important theoretically if we try to understand structure and function of pelagic communities. Many pelagic species derive most of their carbon from emergent benthopelagic zooplankton by capturing small numbers of relatively large taxa, such as decapods and mysids [32]. The predation impact of benthopelagic animals on the pelagic zooplankton (33–154% of the zooplankton production) may be high and suggests an important role for this group in the water column [33]. Estimation of the contribution that benthopelagic animals make to the near-shore plankton communities in the Black Sea is the fourth objective of the paper.

Shelf benthopelagic communities are of a particular practical interest, especially in the areas of increasing anthropogenic pressure including fishing activity, oil spills and marine reserves. Daytime composition of the Black Sea plankton has been studied regularly over 80 years [34]–[48]. However, almost nothing is known about plankton composition and dynamics at night when numerous benthopelagic animals are expected to swim in the water column. Intensive studies in 1960 s were focused on the surface water layer (0–45 cm) at night only. These observations showed that the ecological group called “benthohyponeuston” ( = benthopelagic plankton ascending to surface) make a significant contribution to the plankton abundance and biomass in the near-surface layer (“neustal”) at night [49]–[53]. However, current knowledge is based on historical studies only.

Surprisingly, for such an explored area as the Black Sea, the dynamics of its coastal ecosystems between sunset and sunrise are poorly known. Nothing is known about the benthopelagic component of the near-shore plankton supporting the trophic webs of the fisheries. There is no previous information regarding possible human impacts on benthopelagic communities, and the possible effects of fishing activity, oil spills and marine reserves remain totally unknown. Knowledge of the recent state of the benthopelagic communities will provide a baseline for future surveys aimed at an estimation of increasing anthropogenic pressure on the plankton communities.

## Materials and Methods

### 1. Sampling Area

The Black Sea is one of the largest intercontinental basins (area 436,400 km^2^, depth 2,212 m, volume of 547,000 km^3^). The sea is the largest meromictic basin on Earth. Below ∼100 to 150 m it is anoxic and inhabited only by bacteria.

**Figure 1:**
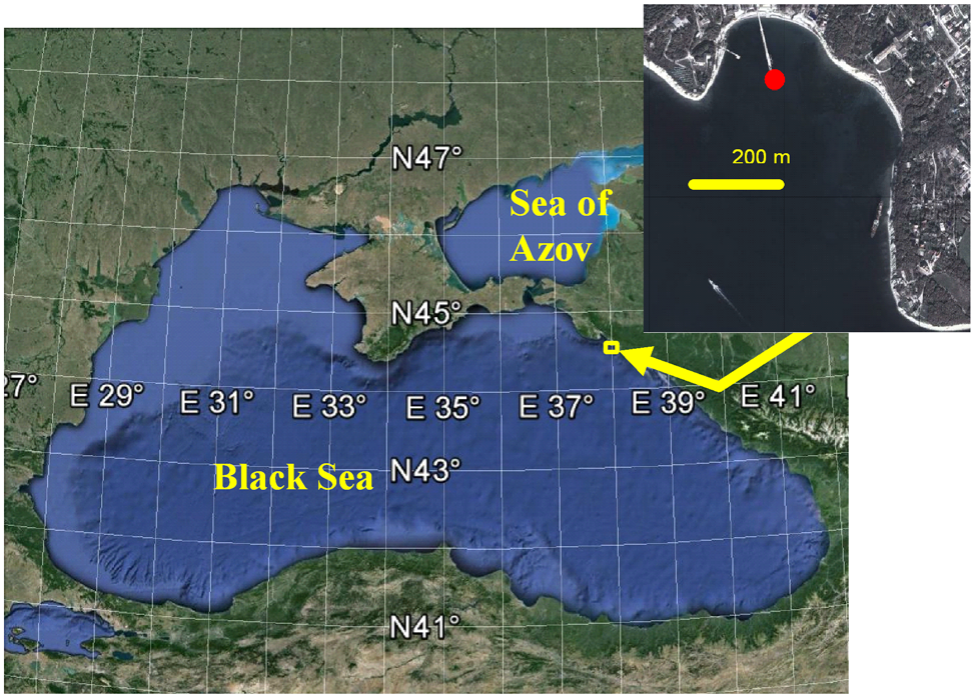
Sampling site (Golubaja Bay). Red circle: sampling site 44°34′31.04″ N, 37°58′45.11″ E, depth 7 m. This figure is similar but not identical to the original image generated by Google, and is therefore for representative purposes only.

Samples were taken in the Golubaja (“Blue”) Bay near Novorossijsk (Fig. 1) between 7 and 10 m deep and characterized by environmental parameters for the Northeast coast of the Black Sea [46]–[47]. The local seafloor is covered with sand interspersed with scattered rocks and algae dominated by *Cystoseira barbata*. This biotope harbored benthopelagic animals by day.

Samples were taken in the Golubaja (“Blue”) Bay near Novorossijsk, GPS coordinates for the sampling site are 44°34′31.04″ N, 37°58′45.11″. Institute of Oceanology, Russian Academy of Sciences, responsible for this area, issued the permit for this location. The field studies did not involve endangered or protected species.

### 2. Sampling Approach

We took two sets of samples: day-and-night and seasonal. Day-and-night samples were to determine the diurnal dynamics of benthopelagic animals (precision 3 hours) and its possible variations at different seasons. Day-and-night samples were taken at all seasons with use of the same schedule: they started at 09∶00, were taken every 3 hours, and ended next day at 12∶00 (local time) (Table 1).

**Table 1:** Day-and-night stations.

Date	Surfacetempe-rature, °C	Airtempe-rature, °C	Sunset	Sunrise	Duration ofnight, hour:min	No of samples
July 15–16, 1996	–	26.7	21∶11	5∶58	8: 47	9
July 16–17, 1996	–	26.6	21∶11	5∶59	8∶48	8
July 17–18, 1996	–	26.2	21∶10	6∶00	8∶50	8
September 1–2, 1997	22.0	22.6	20∶07	6∶51	10∶44	10
September 4–5, 1997	18.6	13.6	20∶02	6∶55	10∶53	10
September 7–8, 1997	19.0	16.3	19∶56	6∶58	11∶02	10
August 26–27, 1998	21.4	21.2	20∶18	6∶44	10∶26	10
August 30–31, 1998	22.3	21.2	20∶11	6∶49	10∶38	10
September 3–4,1998	16.3	16.1	20∶03	6∶54	10∶51	10
August 30–31, 1999	22.0	21.2	20∶11	6∶49	10∶38	10
September 3–4, 1999	22.8	21.4	20∶03	6∶54	10∶51	10
September 7–8, 1999	23.0	21.0	19∶56	6∶58	11∶02	10
October 18–19, 1999	20.1	10.0	18∶40	7∶48	13∶08	10
October 24–25, 1999	17.2	8.2	18∶31	7∶56	13∶25	10
December 8–9, 1999	11.6	10.5	16∶48	7∶53	15∶05.	10
February 25–26, 2000	7.5	5.0	18∶09	7∶12	13∶03.	10
March 2–3, 2000	8.2	8.1	18∶17	7∶02	12∶45.	10
June 5–6, 2000	19.6	20.1	21∶10	5∶44	8∶34.	10
July 17–18, 2000	25.0	24.0	21∶10	6∶00	8∶50	10
July 24–25, 2000	25.6	23.2	21∶04	6∶07	9∶03	10
September 4–5, 2000	24.1	21.0	20∶00	6∶56	10∶56	10
September 11–12, 2000	23.0	20.0	19∶47	7∶04	11∶17	10
July 19–20, 2001	26.7	26.0	21∶09	6∶01	8∶52	10
October 15–16, 2001	20.6	11.8	18∶46	7∶44	12∶58	20
September 13–14, 2006	22.3	18.3	19∶45	7∶05	11∶20	22
August 29–30, 2007	26.6	21.7	20∶12	6∶48	10∶36	26
September 18–19, 2007	21.4	15.9	19∶35	7∶11	11∶36	28

Seasonal samples were taken to determine the seasonal dynamics of benthopelagic animals throughout a year. Seasonal samples were taken using the same schedule: they started at 00.00 A.M. and finished at 00.15 A.M. every 10 days during the season 1999–2000, except the period December-February when samples were rare due to winter storms.

Plankton was caught at a distance of 170 m from the coast with a Judey net (mouth area 0.1 m^2^, mesh size 180 µm), towed at 50 cm s^−1^ from 6.5 m depth obliquely to the surface. All samples were accompanied by measurements of surface temperature with Shpindler thermometer and meteorological data.

A total of 321 samples (Table 1) were made from 1996 to 2007 to indicate benthopelagic animals.

### 3. Treatment of Samples

Samples were preserved in 4% seawater-formaldehyde solution and identified to species level where possible using a stereomicroscope. Species were identified with use of [54]–[56], recent taxonomy was checked with use of Word Register of Marine Species [http://www.marinespecies.org]. For each taxon the number of specimens in the sample and individual sizes with the precision of 0.1 mm were recorded. On the basis of this primary dataset the individual weights, species abundance and biomass, and the total abundance and biomass were calculated with use of the Plankton samples treatment program PLANKTY [57]. When abundances and biomass (individuals and wet weight per m^3^) were calculated, the filtration coefficient was assumed to be 1.

### 4. Statistical Analyses and Data Availability

In order to test seasonal variations of the contribution that benthopelagic animals make to the total plankton biomass we used two non-parametric tests: (1) the Kruskal–Wallis one-way analysis of variance by ranks for combination of four seasons and (2) the Mann–Whitney U test for each pair of seasons.

To examine the possible correlation between temperature and biological parameters numbers, we used the Pearson product-moment correlation coefficient.

Information about sampling efforts is published in tables and figures of this paper. Primary field information may be sent on request and will further be deposited in the database www.inviders.ocean.ru recently organized by the laboratory of the first author.

## Results

### 1. Species Composition

Sixty nine species were recorded which repeatedly (more then 10 times) occurred in the water column at night and were absent during the day (Table 2). We consider these species to be diurnally-migrating truly benthopelagic (peracaridians, late decapod larvae at the megalopa stage). In contrast, the animals occurring in the water column during both by day and at night were treated as pelagic (calanids, comb-jellies) or meroplanktonic (larvae of bivalves, gastropods).

**Table 2:** List of benthopelagic animals.

Dominant species	Common species	Rare species
**Amphipoda**		
*Apherusa bispinosa*	***Ampithoe gammaroides***	*Perioculodes longimanus*
***Echinogammarus olivii***	***Ericthonius difformis***	***Stenothoe monoculoides***
***Hyale pontica***	*Caprella acanthifera*	***Melita palmata***
*Atylus guttatus*	***Chaetogammarus ischnus***	***Microprotopus longimanus***
***Gammarus insensibilis***	*Microdeutopus gryllotalpa*	***Megaluropus massiliensis***
*Dexamine spinosa*		***Jassa ocia***
		***Chelura terebrans***
		***Amphithoe ramondi***
		***Crassicorophium crassicorne***
**Mysidacea**		
*Siriella jaltensis*	***Diamysis mecznicowi***	***Hemimysis lamornae pontica***
	***Mesopodopsis slabberi***	*Paramysis (Occiparamysis) agigensis*
	***Leptomysis lingvura***	
**Isopoda**		
	*Idotea balthica*	***Limnoria tuberculata***
	***Idotea ostroumovi***	*Sphaeroma serratum*
	**Dynamene bidentata**	*Lekanesphaera hookeri*
	***Elaphognathia bacescoi***	*Eurydice pontica*
**Cumacea**		
***Nannastacus unguiculatus***	*Bodotria arenosa mediterranea*	***Iphinoe tenella***
	*Cumella (Cumella) pygmaea euxinica*	***Iphinoe elisae***
	*Cumella (Cumella) limicola*	*Pseudocuma (Pseudocuma) longicorne*
**Tanaidacea**		
		*Leptochelia savignyi*
		***Tanais dulongii***
**Decapoda (late larvae at the megalopa stage)**		
***Hippolyte inermis***	***Hippolyte leptocerus***	***Alpheus dentipes***
***Diogenes pugilator***	*Palaemon elegans*	***Processa edulis edulis***
***Pestarella candida***	***Upogebia pusilla***	***Lysmata seticaudata***
***Xantho poressa***	***Necallianassa truncata***	*Crangon crangon*
	***Athanas nitescens***	***Clibanarius erythropus***
	***Pisidia longimana***	
**Polychaeta**		
	***Exogone naidina***	***Nephtys hombergii***
	***Salvatoria limbata***	*Micronephtys stammeri*
	***Syllis*** ** sp.**	***Prionospio cirrifera***
		***Amblyosyllis formosa***
		*Sphaerosyllis bulbosa*
		***Eulalia viridis***
		***Dorvillea rubrovittata***
		***Nereis rava***

Bold type: first record of diurnal vertical migrations.

Our data show that the benthopelagic fauna of the Northeast coast of the Black Sea is represented by: Amphipoda (20 species), Mysidacea (6 species), Cumacea (7 species), Isopoda (8 species), Anisopoda (2 species), Decapoda (15 species), and Polychaeta (11 species). All recorded benthopelagic species were divided into 3 groups: (1) dominant, regularly recorded and composing at least 50% of the total plankton abundance or biomass during period of their maximal concentration; (2) rare species represented by less than 20 individuals in all the samples and (3) common species (regularly recorded and never composing 50% of the total plankton abundance or biomass). Twelve species dominated: 6 amphipods (*Apherusa bispinosa, Echinogammarus olivii, Hyale pontica, Atylus guttatus, Gammarus insensibilis*, *Dexamine spinosa*), 1 mysid (*Siriella jaltensis), 1* cumacean *(Nannastacus unguiculatus),* and 4 decapods (late larvae of *Hippolyte inermis, Diogenes pugilator, Pestarella candida, Xantho poressa).*

### 2. Diurnal Variations of the Biomass, Abundance, and Composition

**Figure 2:**
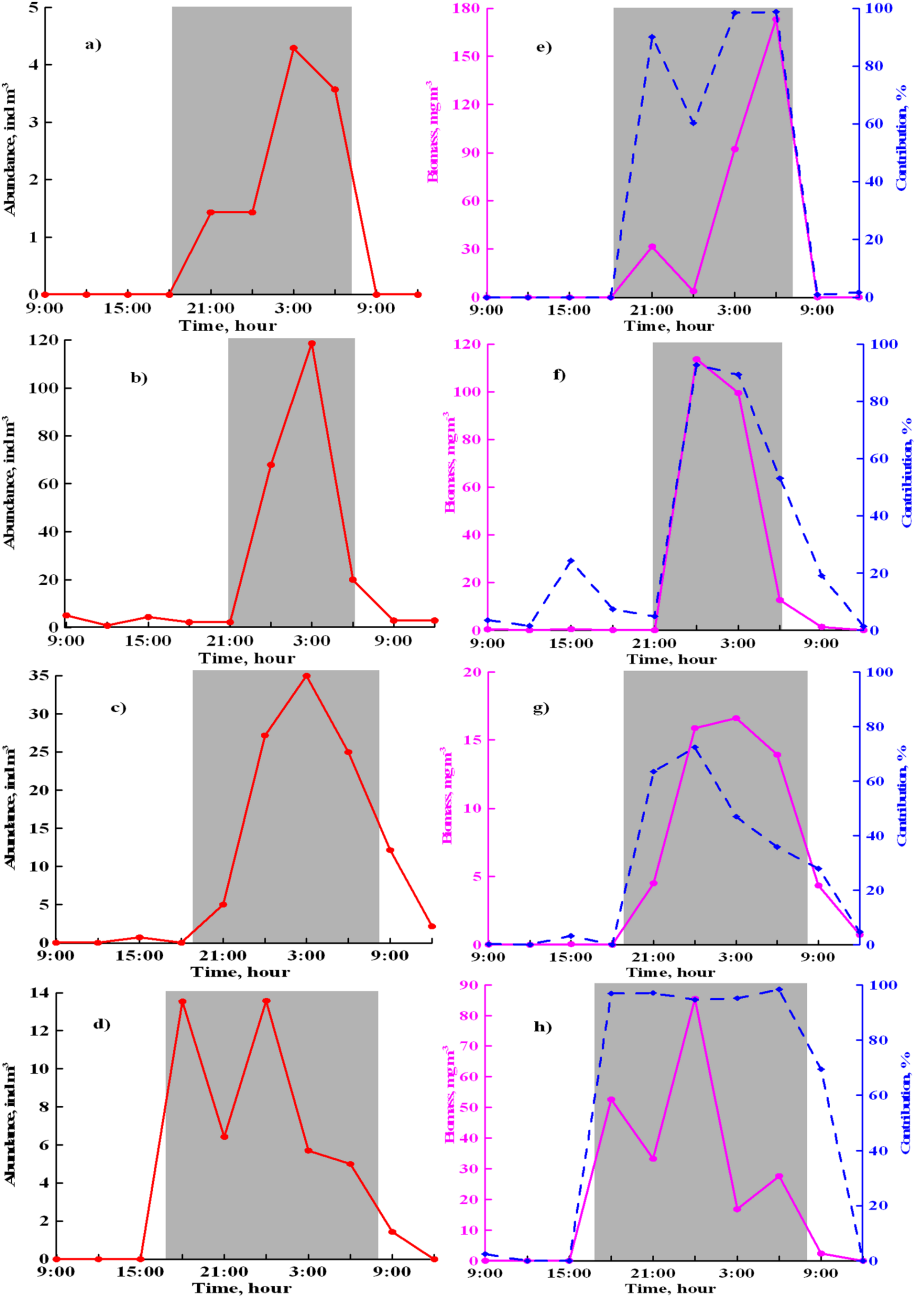
Overnight dynamics of abundances (a–d, red line), biomass (e–h, pink line), and contribution to the total plankton biomass (e–h, blue line) of benthopelagic animals: March 2–3, 2000 (a, e), July 24–25, 2000 (b, f), October 18–19,1999 (c, g); December 8–9, 1999 (d, h).

During all seasons, the main bulk of the benthopelagic animals ascended through the water column 1 hour after sunset (Fig. 2). Total abundances and biomass were maximal around midnight and later decreased; around 1 hour before sunrise most benthopelagic animals disappeared from the water column again. During cloudy, windy and/or foggy weather benthopelagic animals stayed in the water column longer: they left the sea-floor ½–1 hour earlier and returned ½–1 hour later. Darkness hours increase considerably over the site in winter and there is a synchronously-increased period when benthopelagic animals occurred in the water column: from 8–10 hours in summer to 13–15 hours in the winter (Fig. 2).

**Figure 3:**
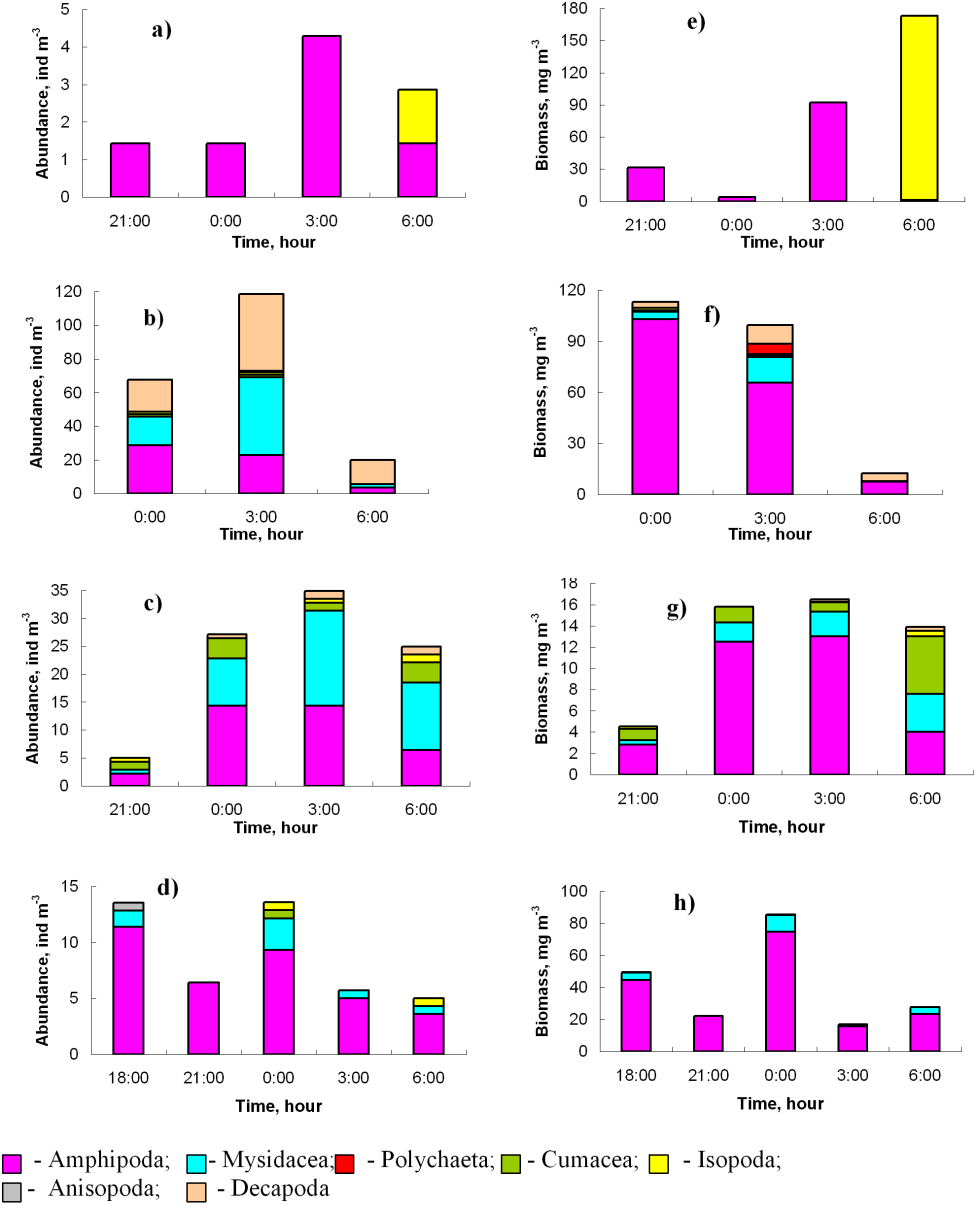
Typical overnight dynamics of abundances (a–d) and biomass (e–h) of the major benthopelagic taxa: March 2–3, 2000 (a, e), July 24–25, 2000 (b, f), October 18–19,1999 (c, g); December 8–9, 1999 (d, h).

Amphipods significantly contributed to the total abundances of the benthopelagic plankton across the dark period, especially in the first half-night, and dominated in the total biomass throughout the dark period (Fig. 3). Juveniles of *Apherusa bispinosa*, *Dexamine spinosa*, *Ericthonius difformis*, as well as juveniles and adults of *Caprella acanthifera ferox* and *Microdeutopus gryllotalpa* occasionally occurred in the water column by day.

Decapods were present in the water column overnight dominating the total abundance and biomass near midnight and sometimes later during warm periods (Fig. 3). Late larvae of *Athanas nitescens*, *Diogenes pugilator, Pisidia longimana*, *Hippolyte inermis* were occasionally present in the daytime. Mysids occurred in the water column overnight except the period February-June when were either present during first half-night or absent.

Cumaceans were present during all dark periods, being abundant in summer and autumn, occasional in the winter but very rare in spring (Fig. 3).

Isopods occurred in the water column overnight regularly, except late winter when they were absent (Fig. 3). Juveniles of *Idotea baltica basteri* and *Dynamene bidentata* sporadically occurred in the water column by day.

Polychaetes occurred in the water column for short periods of time and were present only in summer (Fig. 3). Small syllids *Salvatoria limbata* were the first benthopelagic animals to migrate from the bottom, usually 20–30 minutes after sunset. Heteronereid forms and juveniles of *Nereis rava* were recorded near the midnight.

### 3. Seasonal Variations of the Biomass, Abundance, and Composition

**Figure 4:**
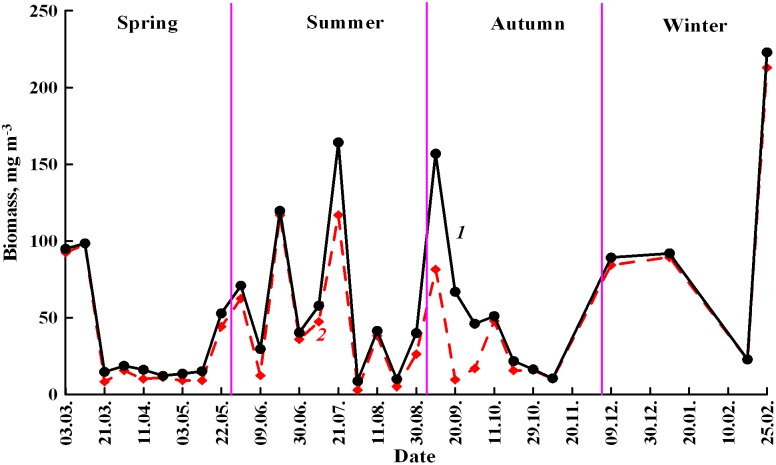
Seasonal variations of biomass of the total zooplankton (*black circles*) and of the benthopelagic component (*diamonds*).

During all seasons, benthopelagic animals compose more than 50% of the total zooplankton biomass (Fig. 4). Their contribution to the total plankton biomass seasonally changed (Fig. 5.1) being maximal during winter and spring. In summer, their dominance decreased and was minimal during autumn. In the winter, plankton consisted almost exclusively of the benthopelagic animals (mainly Amphipods). The share of benthopelagic plankton decreased in the progression: winter-spring-summer-autumn due to increasing growth and reproduction of the pelagic animals and meroplanktonic larvae. We observe synchronous increase of the standard deviations (SD) due to increased variability of the pelagic plankton and meroplankton biomass.

**Figure 5:**
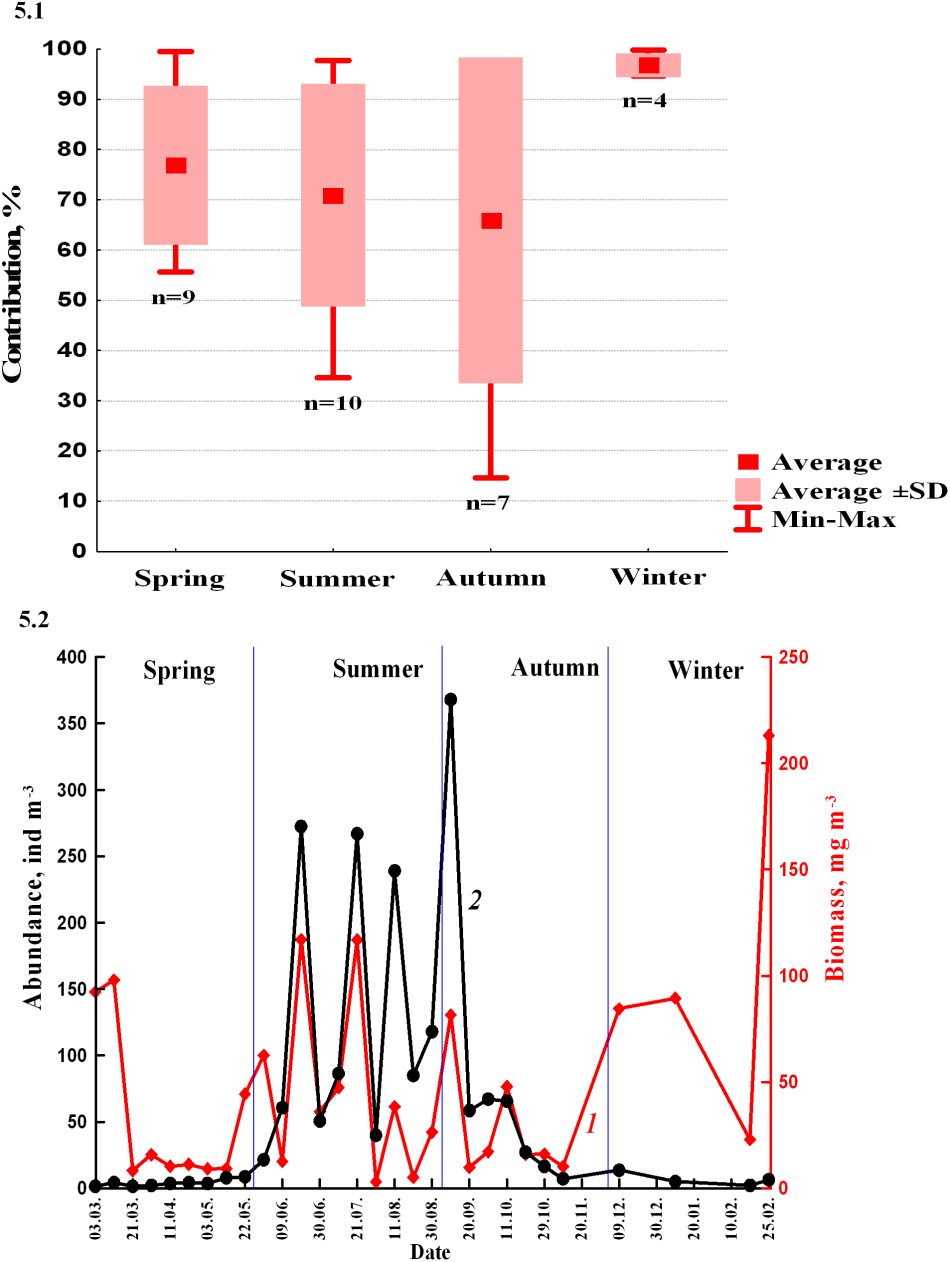
Contribution (%) that the benthopelagic animals made to the total biomass of the zooplankton during the season 1999–2000 in the Golubaja Bay (5.1.); n: number of samples taken during the selected season. Inter-seasonal variations of abundances (black line 2) and biomass (red line 1) of the benthopelagic animals at midnight during the period 1999–2000 (5.2).

The Kruskal–Wallis one-way analysis of variance by ranks shows that the contribution of the benthopelagic animals to the total plankton biomass differs during four seasons at the p level 0.1 (df = 29). Further use of the Mann-Whitney U test indicates more statistically significant level between autumn and winter (p = 0.05, df = 8) and between winter and spring (p = 0.05, df = 12). The Mann-Whitney U test fails for the pair summer-autumn due to increased variability of the non-benthopelagic component at these periods and increased standard deviation.

The highest biomass of the benthopelagic plankton was recorded during winter and early spring (100±61 mg m−^3^, n = 6) when adult amphipods dominated (Fig. 5.2). This period was followed by the period of the lowest biomass (from the middle of March to the middle of May: 10±3 mg m^−3^, n = 6). From the middle of May to the middle of September the biomass rose (49±39 mg m^−3^, n = 12) and from the end of September to the end of November the biomass fell again (19±14 mg m^−3^, n = 6).

Seasonal changes of abundance differed from those of biomass, being more gradual (Fig. 5.2). In contrast to biomass, abundance was minimal during winter (6±4 ind m^−3^, n = 6) and spring (5±3 ind m^−3^, n = 6). Highest abundances were recorded during summer (146±118 ind m^−3^, n = 6) and autumn (47±23 ind m^−3^, n = 6) when most benthopelagic species reproduced. Since at these periods they were represented by larvae and juveniles, the increase in abundance did not lead to significant increase in the biomass.

**Figure 6:**
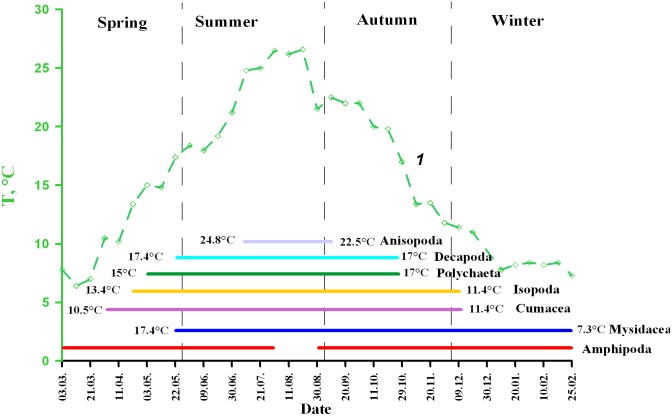
Presence of the major taxa of the benthopelagic animals in the water column of the Golubaja Bay during the season 1999–2000. *1*– surface temperature.

Year-round observations showed that the major taxa of benthopelagic animals appeared in the water column depending on temperature (Fig. 6). Amphipods were present throughout the year excluding the middle of August when water temperature was maximal (26–27°C). At this temperature the species stop diurnal migrations into the water column. Most taxa appeared in and disappeared from the water columns at similar temperatures (Fig. 5). Only mysids appeared at higher temperatures.

Further analysis of correlation between temperature and available integral characteristics of the benthopelagic plankton revealed statistically significant correlations (p≤0.05, df = 29 for all). Positive correlation between water temperature and

- share of decapods in the total abundance (R = 0.76) and biomass (R = 0.62) of the benthopelagic plankton.
- number of benthopelagic species (R = 0.75).
- abundances of benthopelagic plankton (R = 0.60).

Negative correlation between water temperature and

- share of amphipods in the total abundance (R = −0.86) and biomass (R = −0.70) of the benthopelagic plankton.
- share of benthopelagic animals in the total plankton biomass (R = −0.48).
- biomass of benthopelagic plankton (R = −0.30).

**Figure 7:**
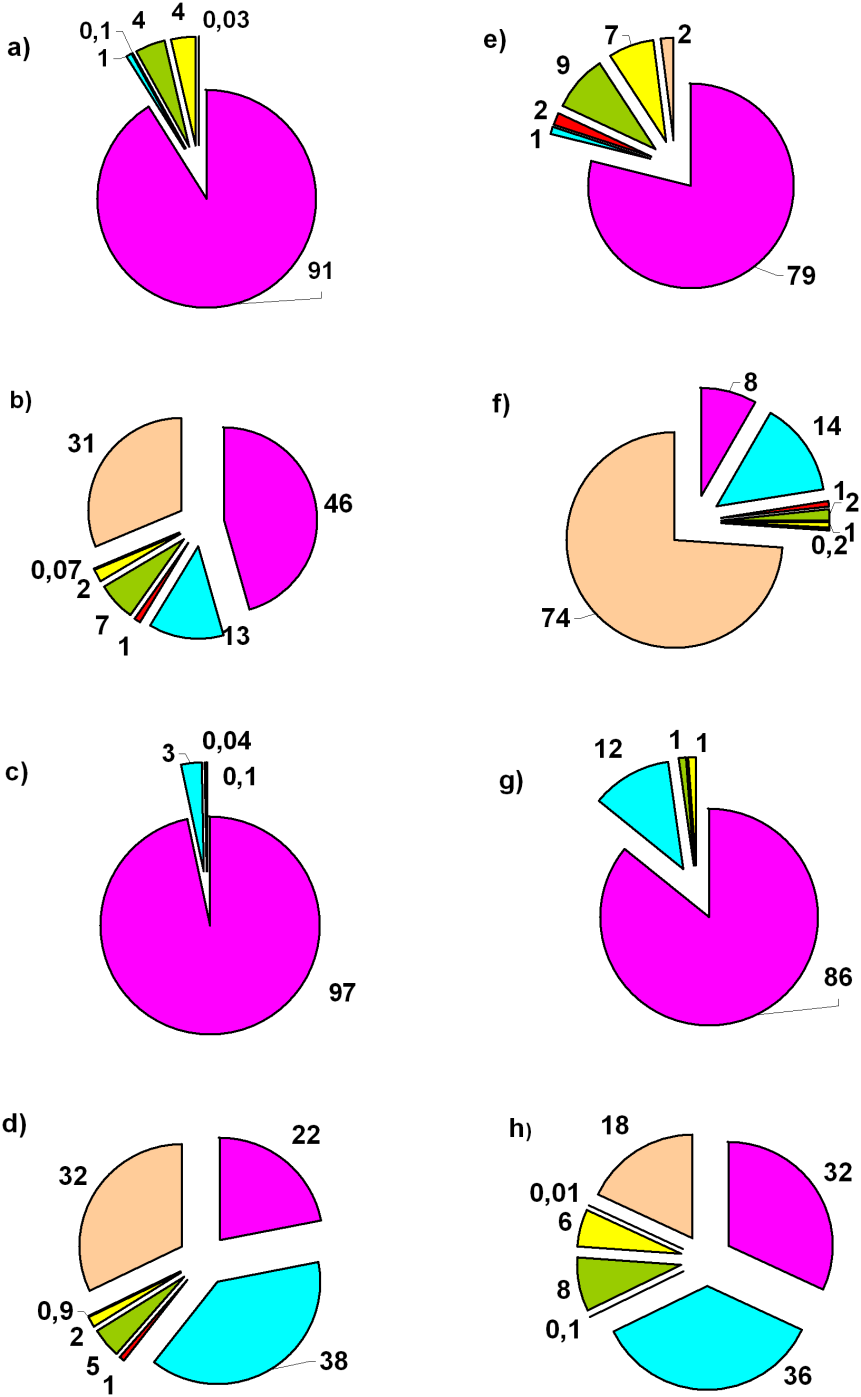
Average contribution (%) that major taxa made to the total biomass (a-d) and abundances (e–h) of the benthopelagic animals during various seasons of 1999–2000 in the Golubaja Bay: March 2–3, 2000 (a, e), July 24–25, 2000 (b, f), October 18–19, 1999 (c, g); December 8–9, 1999 (d, h). Colour legend as in Fig. 3.

The contribution which major benthopelagic taxa made to the total abundances and biomass varied with season (Fig. 7). Amphipoda made the main contribution (32–97% in different seasons) to the total biomass of the benthopelagic animals. The second important group, Mysidacea, composed 1–36% of the total biomass (during autumn even more than the amphipod). Decapoda (18–31%) and Cumacea (7–8%) were less important.

In terms of abundance (Fig. 7), Amphipoda dominated during winter and spring (79% and 86% of the total abundance of benthopelagic animals, respectively). During summer and autumn the dominant groups were Decapoda (74% and 32%, respectively) and Mysidacea (14% and 38%, respectively).

## Discussion

### 1. Species Composition

The semiclosed meromictic Black Sea harbours an unexpected diverse benthopelagic fauna that includes 12 species dominating in plankton communities at night. Zaitsev [48]–[49] and Zakutsky [50]–[51] recorded a total of 23 zooplankton species in the Black Sea that appeared in the water column at night only and thus might be treated as benthopelagic.

Our studies have significantly extended this list and showed that the benthopelagic fauna at the studied site (69 species) is comparable to that occurring above the continental shelf and upper slope in similar geographic areas. In the Mediterranean, 62 taxa were found in Ebro River Delta, Spain [13], 86 taxa were reported from the Gulf of Castellammare, Italy [58], and 96 taxa occurred in Heraklion Bay, Crete [11]. Western European shelves harbour similar number of benthopelagic taxa: 52–97 taxa in Arcachon Bay, Biscay [59] and 118 taxa in Gullmarfjord, the North Sea [60].

More distant tropical coral reef and mangroves communities, very rich and specious in general, contain the same order of magnitude (tens) of benthopelagic taxa [16]–[22]. Given a single site studied in the Black Sea, we may expect that the species list for this basin is longer and the benthopelagic fauna is at least as diverse as in similar geographic areas. En mass, we may suppose that within temperate and tropical latitudes the benthopelagic biodiversity does not significantly depend on geographic area and generally comprises 50–100 taxa. This hypothesis should be tested in future studies embracing a wider range of comprehensively studied biotopes.

### 2. Diurnal Variations of the Biomass, Abundance, and Composition

Total abundances of the benthopelagic animals found in the Black Sea (20–370 ind m^−3^in summer) have same order of magnitude as those reported for similar shelf depths. Recorded abundances range from 5–7 ind m^−3^ (the English Channel [61] and the Dutch Delta at 5–10 m [62]) and 10–14 ind m^−3^ (Oslofjord and the west coast of Norway at 22–100 m [63]) to 40 ind m^−3^ (the Kiel Bay at 30 m [64] and the Gulf of St. Lawrence at 30 m [65]). Higher abundances were found in areas influenced by river discharges: up to 65 ind m^−3^ of peracarids off the Ebro Delta at 47–61 m [13] and 389 ind m^−3^ in the Bay of Morlaix [66]. Thus, the total abundances of the benthopelagic plankton at the studied site fall inside the known limits and correspond to levels of organically-enriched biotopes near river discharges. One of possible causes of this phenomenon may be related to the nearby mouth of a small river, the Ashamba, whose impact to the near-shore communities still remains to be understood.

The general pattern of the benthopelagic abundance and biomass is characterized by two main peaks in summer and in the winter. The former is associated with reproductive cycles and has been recorded also in the Mediterranean [13]. The latter may be specific for the shallow sites of intercontinental basins where the impact of terrigenous organic matter is significant.

Overall, the benthopelagic animals occupied the water column during the whole dark period from sunset to sunrise accounting for more than 50% of the total plankton biomass most of this time. The migrations start approximately 1 hour after sunset when the surface illumination decreases to about 1 lux [8]–[9]. Similar level of illumination before sunrise makes benthopelagic animal settle to the sea-floor. Swimming activity varies individually and occasionally amphipod juveniles (*Apherusa bispinosa*, *Dexamine spinosa*, *Ericthonius difformis*) and even adults (*Caprella acanthifera ferox, Microdeutopus gryllotalpa*) may occur in the water column by day – the phenomenon previously reported [8], [26].

As soon as the sun sets, the coastal plankton communities drastically change. Numerous taxa ascend to the water column from the sea-floor. Appearance of these taxa is driven by biological factors. Most captured polychaetes bear eggs and are probably spawning. Amphipods and cumaceans are represented by mature males and females which likely couple. Juveniles of isopods and amphipods may act as dispersal stages. Stomachs of late decapod larvae and mysids are full of plankton and indicate that they mainly feed. Overnight the composition of phytoplankton significantly changes and these changes are also modulated by the seasonal variations.

### 3. Seasonal Variations of the Biomass, Abundance, and Composition

The seasonal dynamics of the benthopelagic abundances and biomass may be related to a number of environmental variables (temperature, salinity, stratification, turbidity, oxygen concentration). Maximum abundance may appear from early summer in shallow waters [62] to late autumn [67]). Possible factors determining the maxima have rarely been considered, though in general, a link between benthopelagic abundance and food supply has been indicated [13].

Trophic aspects may be important in the seasonal dynamics of the Black Sea benthopelagic fauna. Near the northeast coast both concentration of chlorophyll *a* in surface waters and primary production are quite variable, generally ranging from 0.2 to 1.0 mg m^−3^and from 0.2 to 0.6 gC m^−2^ day^−1^, respectively [68]. Average values, however, do not change seasonally and thus can not cause significant variations of the benthopelagic abundance and biomass. Allochthonous organic matter washed off the shores by winter storms and brought by small rivers may be significant contributors but their impact is local and still waits for future research to estimate.

Temperature is one of the most important seasonal factors that control biological processes within plankton populations and communities.

The share of decapods in the total abundance and biomass of the benthopelagic plankton is positively correlated with temperature. In the warm reproductive period decapods significantly contribute to the total benthopelagic abundances and biomass. Conversely, in the cold period decapods are adult and only occasionally occur in the plankton.

Conversely, the share of amphipods in the total abundance and biomass of the benthopelagic plankton is negatively correlated with temperature. Amphipoda is the only taxon abundant both in warm and cold seasons. In the winter and spring they account for the main bulk of the benthopelagic biomass, whilst in summer and autumn amphipods are overwhelmed by various taxa of seasonally reproduced benthopelagic animals. The sharp decrease of amphipod density and contribution in summer and autumn is probably related to the general biological cycle of peracarids, with a massive post-reproductive mortality of populations in late summer–early autumn after successive juvenile releases [13], [67], [69]–[70].

Amphipods and decapods are seasonal counterparts in the benthopelagic communities, the former being the leading group in cold period, while the latter significantly contributes in summer-autumn.

Abundances and number of benthopelagic species are positively correlated with temperature. The main contribution to this phenomenon is made by numerous decapod taxa. Decapods are specious group of benthopelagic animals with high abundances. They sufficiently enrich the benthopelagic diversity and abundances during period of reproduction in summer and autumn.

The proportion of benthopelagic animals in the total plankton biomass and the biomass of benthopelagic plankton are negatively correlated with temperature. During summer-autumn the non-benthopelagic component dominated by copepods with well-marked seasonal cycles becomes a very important component. During warm periods this component reaches high biomass, while benthopelagic peracarids are not dense due to post-reproductive mortality and juvenile releases. Conversely, benthopelagic animals, mainly amphipods, demonstrate high biomass at cold seasons when they are represented by adults. It is in the winter when benthopelagic animals may use organic matter more efficiently because of low number of pelagic competitors.

Stratification hardly influences seasonal dynamics of the benthopelagic animals at the site, as the seasonal thermocline occurs at depth 10–30 m, and the water column at studied site is situated within the mixed layer across the year.

Seasonal changes in the abundances and biomass of benthopelagic taxa may also be a result of horizontal migrations from shallow to deeper waters. For example, mysids may avoid unfavorable winter conditions, e.g. low temperatures, winter storms. Such migrations have been reported from field and laboratory observations for mysids [71]–[73]. Conversely, the amphipods in same period may be attracted by large amounts of high-quality organic matter washed away from the shores. Similar migrating behaviour associated with wood availability has been recorded for the peracarids in the Bay of Biscay [71] and near Crete [11]. Conversely, mobile crustaceans [74]–[75] can migrate away from unfavorable environmental conditions.

As a general conclusion, benthopelagic fauna of the northeast shelf of the Black Sea is rich and experiences clear diurnal and seasonal changes. These dynamics are a result of environment changes coupled with behaviour and life cycles of biota. The coastal Black Sea plankton drastically changes at night when the structure of the plankton communities depends on benthopelagic animals (in contrast to the daytime when holoplankton dominates). Both night and daytime samples are strongly recommended for the adequate description of the plankton communities of the semiclosed and other seas. The data presented were obtained at shallow depths in a single bay of the Black Sea. Future studies should show what happens above greater depths.

## References

[1] Armi L, Millard RC Jr (1976) The bottom boundary layer of the deep ocean. J Geophys Res 81: 4983–4990.

[2] Vereshchaka AL (2000) The deep-sea benthopelagic zone: life near the sea-floor. Moscow, Nauchnyj Mir 240 p [in Russian].

[3] Grice GD, Hulsemann K (1970) New species of bottom-living calanoid copepods collected in deep water by the DSRV ALVIN. Bull Mus Comp Zool 139: 185–227.

[4] Grice GD (1972) The existence of a bottom-living calanoid copepod fauna in deep water with descriptions of five new species. Crustaceana: 219–242.

[5] Marshall NB, Merrett NR (1977) The existence of a benthopelagic fauna in the deep sea. In: Angel M (Ed) A Voyage of Discovery: George Deacon 70th Anniversary, Pergamon Press Ltd, Oxford, p 483–497.

[6] Wishner KF (1980) The biomass of the deep-sea benthopelagic plankton. Deep Sea Res Pt. A 27: 203–216.

[7] Vereshchaka AL, Vinogradov ME (2002) Three-dimensional view of the Atlantic abyssal benthopelagic vent community. Cah Biol Mar 43(3–4): 303–306.

[8] Macquart-Moulin C (1984) La phase pelagique nocturne et les comportements migratoires des amphipodes benthiques (Mediterranee, Nord-Occidentale). Tethys 11: 171–196.

[9] Macquart-Moulin C, Maycas E (1995) Inshore and offshore diel migrations in European benthopelagic mysids, genera *Gastrosaccus*, *Anchialina* and *Haplostylus* (Crustacea, Mysidacea). J Plankt Res 17: 531–555.

[10] Vereshchaka AL (1995) Macroplankton in the near-bottom layer of continental slopes and seamounts. Deep-Sea Res Pt I 42: 1639–1668.

[11] Fossa JH (1985) Near-bottom vertical zonation of deep-living hyperbenthic mysids (Crustacea: Mysidacea). Sarsia 70: 297–307.

[12] Kaartved S (1989) Retention of vertically migrating suprabenthic mysids in fjords. Mar Ecol Progr Ser 57: 119–128.

[13] Cartes JE, Papiol V, Palanques A, Jorge G, Demestre M (2007) Dynamics of suprabenthos off the Ebro Delta (Catalan Sea: western Mediterranean): Spatial and temporal patterns and relationships with environmental factors. ECSS 75: 501–515.

[14] Dauvin JC, Desroy N, Denis L, Ruelle T (2008) Does the *Phaeocystis* bloom affect the diel migration of the suprabenthos community? Mar Poll Bull 56: 77–87. 10.1016/j.marpolbul.2007.09.041 18023458

[15] Hammer RM, Zimmerman RC (1979) Species of demersal zooplankton inhabiting kelp forest ecosystem off Santa Catalina Island, California. Bull South Cal Acad Sci 78: 199–206.

[16] Kringel K, Jumars PA, Holliday DV (2003) A shallow scattering layer: High-resolution acoustic analysis of nocturnal vertical migration from the seabed. Limnol Oceanogr 48: 1223–1234.

[17] Yahel R, Yahel G, Berman G (2005) Diel pattern with abrupt crepuscular changes of zooplankton over a coral reef. Limnol Oceanogr 50: 930–944.

[18] Carleton JH, Hamner WM (2007) The hyperbenthic plankton community: composition, distribution, and abundance in a coral reef lagoon. Mar Ecol Prog Ser 336: 77–88.

[19] Melo PA, Silva TA, Neumann-Leitão S, Schwambornc R, Gusmão LM, et al (2010) Demersal zooplankton communities from tropical habitats in the southwestern Atlantic. Mar Biol Res 6: 530–541.

[20] Yoshida T, Tan MK, Tan HS, Othman BH (2012) Substrate complexity and albedo preference of reef. In: Proceedings of the 12th International Coral Reef Symposium, Cairns, Australia, 9–13 July 2012, p 1–4.

[21] Robertson AI, Dixon P, Daniel PA (1988) Zooplankton dynamics in mangrove and other nearshore habitats in tropical Australia. Mar Ecol Progr Ser 43: 139–150.

[22] Robertson AI, Duke NC (1987) Mangroves as nursery sites: comparison of the abundance and species composition of fish and crustaceans in mangroves and other nearshore habitats in tropical Australia. Mar Biol 96: 193–205.

[23] Williams AB (1972) Bynum (1972) A ten-year study of meroplankton in North Carolina estuaries: Amphipods. Chesapeake Sci 44: 171–187.

[24] Grant J (1980) A flume study of drift in marine infaunal amphipods. Mar Biol 56: 79–84.

[25] Greenwood JG, Greenwood J, Skilleter GA (2002) Comparison of demersal zooplankton in regions with differing extractive-dredging history, in the subtropical Brisbane River estuary. Plankt Biol Ecol 49: 17–26.

[26] Tully O, McGrath D (1987) The status of *Idotea metallica* Bosc in Irish waters. Irish Nat J 22: 190–200.

[27] Tully O, Ceidigh P (1986) The ecology of *Idotea* species (Isopoda) and *Gammarus locusta* (Amphipoda) on surface driftweed in Galway Bay (west of Ireland). J Mar Biol Ass UK 66: 931–942.

[28] Tully O, Ceidigh P (1987) Investigation of the plankton of the west coast of Ireland VIII: the neustonic phase and vertical migratory behaviour of benthic Peracaridea in Galway Bay. Proc Roy Irish Acad 87: 43–64.

[29] Vereshchaka AL (2000b) The genus *Sergia*: taxonomy, systematics, and distribution. “Galathea”. Rep 19: 1–207.

[30] Vereshchaka AL (2009) The genus *Sergestes*: taxonomy, systematics, and distribution. “Galathea”. Rep 22: 1–137.

[31] Vinogradov ME (1968) Vertical distribution of the oceanic zooplankton. Acad. Nauk. SSSR Inst. Oceanolog. Moscow. (Transl. by Israel Program for Scientific Transl. Ltd,Jerusalem, Keter Press. 1970).

[32] Pitt KA, Clemen AL, Connolly RM, Thibault-Botha D (2008) Predation by jellyfish on large and emergent zooplankton: implications for benthic–pelagic coupling. ECSS 76: 827–833.

[33] Kouassi E, Pagano M, Saint-Jean L, Sorbe JC (2006) Diel vertical migrations and feeding behavior of the mysid *Rhopalophthalmus africana* (Crustacea: Mysidacea) in a tropical lagoon (Ebrié, Côte d’Ivoire). ECSS 67: 355–368.

[34] Vinogradov KA (1949) On the fauna of worms (Polychaeta). Proc Karadag Biol Sta 8: 1–84 [in Russian].

[35] Vinogradov KA (1931) Some additions to the fauna of Polychaeta in the Black Sea. Proc Karadag Biol Sta 4: 5−22 [in Russian].

[36] Makarov AK (1938) The distribution of some crustaceans (Mysidacea, Cumacea) and clams in estuaries and open lagoons northern Black Sea. Zool Journal 17: 1055−1062 [in Russian].

[37] Nikitin VN (1939) Plankton of the Batumi Bay and its annual change. Pishchepromizdat Press, Moscow [in Russian].

[38] Dolgopolsky MA (1940) Zooplankton of the Black Sea near Karadag. Proc Karadag Biol Sta 6: 57−111 [in Russian].

[39] Klyucharev KV (1952) Materials for the quantitative characterization of the zooplankton of the Black Sea near Karadag. Proc Karadag Biol Sta 12: 78−95 [in Russian].

[40] Kusmorskaya AP (1955) Seasonal and annual changes in the zooplankton of the Black Sea. Proc Soviet Hydrobiol Soc 6: 158−192 [in Russian].

[41] Zakhvatkina KA (1959) Bivalve larvae near Sevastopol, Black Sea region. Proc Sevastopol Biol Sta 11: 108−152.[in Russian].

[42] Chukhchin VD (1960) On detachment *Saccoglossa* (Gastropoda, Opisthobranchia) in the Black Sea. Proc Sevastopol Biol St 13: 89–91.

[43] Kiselyova MI (1967) Addendum to the gastropod fauna of the Black Sea. Zool Journal 46: 764−765 [in Russian].

[44] Greze VN, Baldina EP, Bileva DC (1971) Population dynamics and production of the zooplankton in the main components of the neritic zone of the Black Sea. Bilogiya morya 24: 12−49 [in Russian].

[45] Vinogradov ME, Shushkina EA (1980) Features of the vertical distribution of zooplankton in the Black Sea. In: Vinogradov ME (Ed) Pelagic ecosystems of the Black Sea. Nauka Press, Moscow, p 179−198 [in Russian].

[46] Pasternak AF (1983) Seasonal dynamics of abundance and biomass of zooplankton off the coast of the North Caucasus. In: Seasonal changes in the Black Sea plankton. Nauka Press, p 139−177 [in Russian].

[47] Lebedeva LP, Shushkina EA, Vinogradov ME, Lukasheva TA, Anokhina LL (2003) The multi-year transformation of the structure mesoplankton northeastern coasts of the Black Sea under the influence of invasive ctenophore. Oceanology 43: 710−715 [in Russian].

[48] Vinogradov ME, Lebedeva LP, Vinogradov GM, Musayeva EI, Lukasheva TA, et al (2005) Monitoring of pelagic communities north-eastern Black Sea in 2004, macro-and mesoplankton. Oceanology 45: 381–392.

[49] Zaitsev YP (1961) The near-surface pelagic biocenosis of the Black Sea. Zool Journal 40: 818−825 [in Russian].

[50] Zaitsev YP (1962) Gears and methods for studying hyponeuston. Env Studies 104: 107−117 [in Russian].

[51] Zakutsky VP (1965a) On the concentration of some benthic organisms in the surface layer of the Black and Azov Seas. Oceanology 5: 495−497 [in Russian].

[52] Zakutsky VP (1965b) Daily changes in the composition of the hyponeuston in the Black and Azov Seas. In: First Congress of VGBO. Problems of Hydrobiology. Nauka: p 163 [in Russian].

[53] Zakutsky VP (1965c) To the knowledge of the vertical migrations of some benthic and nectobentic organisms in the Zhebriyanskaja Bay and in the harbor waters near Genichesk. J Hydrobiol 1: 63−66 [in Russian].

[54] Mordukhai-Boltovskoi FD (Ed) (1968) Key to fauna of the Black Sea and the Azov Sea. Kiev: Naukova Dumka 1 437 p [in Russian].

[55] Mordukhai-Boltovskoi FD (Ed) (1969) Key to fauna of the Black Sea and the Azov Sea. Kiev: Naukova Dumka 2 536 p [in Russian].

[56] Mordukhai-Boltovskoi FD (Ed) (1972) Key to fauna of the Black Sea and the Azov Sea. Kiev: Naukova Dumka 2 340 p [in Russian].

[57] Dyakonov VY (2002) Brief description of the program «PLANKTY». In: Sagalevich A (Ed) Oceanographic studies of the Gulf Stream frontal zone: the «Titanic» site. Nauka, Moscow, p 111–114.

[58] Fanelli E, Cartes JE, Badalamenti F, Rumolo P, Sprovieri M (2009) Trophodynamics of suprabenthic fauna on coastal muddy bottoms of the southern Tyrrhenian Sea (western Mediterranean). J Sea Res 61: 174–187.

[59] Sorbe JC (1989) Structural evolution of two suprabenthic soft-bottom communities of the South Gascogne continental shelf. Sci Marina 53: 335–342.

[60] Buhl-Jensen L, Fosså JH (1991) Hyperbenthic crustacean fauna of the Gullmarfjord area (western Sweden): species richness, seasonal variation and long-term changes. Mar Biol 109: 245–258.

[61] Vallet C, Dauvin JC (1998) Composition and diversity of the benthic boundary layer macrofauna from the English Channel. JMBA 78: 387–409.

[62] Mees J, Dewicke A, Hamerlynck O (1993) Seasonal composition and spatial distribution of hyperbenthic communities along estuarine gradients in the Westerschelde. Netherl. J. Aquatic Ecol 27: 2–4.

[63] Hesthagen IH, Gjermundsen B (1978) The replicability of sampling the hyperbenthic region by means of Beyer’s 50 cm. epibenthic closing net. Kieler Meeresforsch 27: 19–26.

[64] Boysen HO (1976) Regional and seasonal differences in the abundance of mysids and decapod larvae in the hyperbenthos of the Kiel Bay. Meeresforsch 25: 54–63.

[65] Huberdeau L, Brunel P (1982) Efficiency and comparative faunistic selectivity of four endo-, epi- and suprabenthic samplers on two bottom types. Mar Biol 69: 331–343.

[66] Dauvin JC, Zouhiri S (1996) Suprabenthic fauna of a dense *Ampelisca* community from the English Channel. JMBA 76: 909–929.

[67] Richoux NB, Deibel D, Thompson RJ (2004) Population biology of hyperbenthic crustaceans in a cold water environment (Conception Bay, Newfoundland). 2. *Acanthostepheia malmgreni* (Amphipoda). Mar Biol 144: 895–904.

[68] Demidov AB (2008) Seasonal dynamics and estimation of the annual primary production of phytoplankton in the Black Sea. Oceanology 48: 664–678.

[69] Cartes JE, Elizalde M, Sorbe JC (2001) Contrasting life-histories, secondary production, and trophic structure of peracarid assemblages of the bathyal suprabenthos from the Bay of Biscay (NE Atlantic) and the Catalan Sea (NW Mediterranean). Deep Sea Res Pt I: 2209–2232.

[70] Richoux NB, Deibel D, Thompson RJ (2004b) Population biology of hyperbenthic crustaceans in a cold water environment (Conception Bay, Newfoundland). 1. *Mysis mixta* (Mysidacea). Mar Biol 144: 881–894.

[71] Beyst B, Buysse D, Dewicke D, Mees J (2001) Surf zone hyperbenthos of Belgian sandy beaches: seasonal patterns ECSS. 53: 877–895.

[72] Dewicke A, Rottiers V, Mees J, Vincx M (2002) Evidence for an enriched hyperbenthic fauna in the Frisian front (North Sea) J of Sea Res. 47: 121–139.

[73] San Vicente C, Sorbe JC (1995) Biology of the suprabenthic mysid *Schistomysis spiritus* (Norman, 1980) in the south-eastern part of the Bay of Biscay. Sci Marina 59: 71–86.

[74] Roast SD, Widdows J, Jones MB (2002) Distribution and swimming behaviour of *Neomysis integer* (Peracarida: Mysidacea) in response to gradients of dissolved oxygen following exposure to cadmium at environmental concentrations. Mar Ecol Progr Ser 237: 185–194.

[75] Hagerman L (1986) Szaniawska (1986) A behaviour, tolerance and anaerobic metabolism under hypoxia in the brackish-water shrimp *Crangon crangon* . Mar Ecol Progr Ser 34: 125–132.

